# Off-Label Use of Ceftazidime/Avibactam for the Treatment of Pan-Drug-Resistant *Klebsiella pneumoniae* in a Neonate: Case Report and Literature Review

**DOI:** 10.3390/antibiotics12081302

**Published:** 2023-08-09

**Authors:** Iliya Mangarov, Ralitsa Georgieva, Valentina Petkova, Irina Nikolova

**Affiliations:** 1 Department of Neonatology, Faculty of Medicine, Pediatric Hospital “Iv. Mitev”, Medical University of Sofia, 1612 Sofia, Bulgaria; rgeorgieva@medfac.mu-sofia.bg; 2 Department of Organisation and Economics of Pharmacy, Faculty of Pharmacy, Medical University of Sofia, 1000 Sofia, Bulgaria; vpetkova@pharmfac.mu-sofia.bg; 3 Department of Pharmacology, Pharmacotherapy and Toxicology, Faculty of Pharmacy, Medical University of Sofia, 1000 Sofia, Bulgaria; inikolova@pharmfac.mu-sofia.bg

**Keywords:** ceftazidime/avibactam, neonate, pan-drug-resistant, *Klebsiella pneumoniae*

## Abstract

Background: *Klebsiella pneumoniae* is among the most common Gram-negative bacteria isolated to neonatal intensive care units (NICU) and one of the leading causes of morbidity and mortality. The ceftazidime/avibactam (CAZ-AVI) combination is approved for infections caused by aerobic Gram-negative organisms. It is licensed for use in infants over 3 months old. There are no safety and efficacy data regarding the administration of CAZ-AVI to infants younger than 3 months, except for a few case reports. Case presentation: This report describes a severely intoxicated 24-day-old, full-term, male neonate transferred to NICU level III from a secondary maternity hospital due to the deterioration of his general condition. On day four of admission, blood culture revealed the pan-drug-resistant (PDR) *K. pneumoniae* ss. pneumoniae, susceptible only to CAZ-AVI, which thus represented the only treatment option. Off-label CAZ-AVI was administered intravenously as a salvage therapy. Conclusions: In healthcare settings, treating resistant *K. pneumoniae* presents serious challenges, especially in NICU patients. The off-label treatment with CAZ-AVI for 17 days was safe and effective in this one-month-old patient. A year later, the patient was healthy with normal cognitive development.

## 1. Introduction

Infections, due to antimicrobial resistant (AMR) pathogens, are perceived as a serious global public health threat, characterized by high morbidity and mortality rates [1,2]. This is especially true in neonatal intensive care units (NICU) due to their limited treatment options compared to older children and adults [3,4]. The surge in using empiric antibiotic therapy, after prompt diagnosis, is unfortunately one of the major causes of AMR [2].

Novel antibiotics and antibiotic combinations exhibit promising activities against resistant pathogens. Ceftazidime/avibactam (CAZ-AVI) was granted marketing approval in Europe in June 2016, and has been on the Bulgarian pharmaceutical market since 2019. CAZ-AVI is licensed for use in adults and children >3 months old. Ceftazidime is a third-generation cephalosporin in clinical use since the 1980s. It acts by inhibiting bacterial enzymes responsible for cell wall synthesis, primarily penicillin-binding proteins (PBPs) 3, which leads to bacterial cell lysis and death. Avibactam is a non-β-lactam, the β-lactamase inhibitor that inactivates a variety of serine β-lactamases (Ambler class A, C and some class D), thus protecting ceftazidime from degradation and preserving its activity against many resistant Gram-negative bacteria. 

The combination, CAZ-AVI, is approved as a last-resort option for the targeted treatment of severe invasive infections caused by laboratory-confirmed aerobic Gram-negative organisms. The antibacterial spectrum covers >99% of Enterobacterales [5]. CAZ-AVI also has activity against ceftazidime-resistant and certain carbapenem-resistant Enterobacterales and *Pseudomonas aeruginosa*, but not strains producing metallo-beta-lactamases (MBLs) (Ambler class B) [5]. The proportion of isolates resistant to CAZ-AVI is relatively low, but emerges worldwide [6,7,8,9,10]. Bacterial resistance mechanisms, that could potentially affect CAZ-AVI, include mutant or acquired PBPs, decreased outer membrane permeability or active efflux to either compound, as well as b-lactamase enzymes being refractory to inhibition by avibactam and able to hydrolyze ceftazidime [11,12]. Resistance is often associated with previous exposure to CAZ-AVI [8].

CAZ-AVI is currently available as a powder for solution for intravenous infusion (2 g of ceftazidime and 0.5 g of avibactam in a fixed 4:1 ratio). Ceftazidime and avibactam are not metabolized and both drugs are eliminated, almost completely unchanged, by the kidneys. The CAZ-AVI dose requires adjustment, according to renal function (estimated CrCl for adults or estimated GFR in pediatric patients). Dosage recommendations are based on the ceftazidime component only. Treatment duration varies according to indication and is usually 7–14 days [13]. In children, CAZ-AVI has been assessed in 128 pediatric patients, in two Phase II trials, for the treatment of complicated urinary tract and intra-abdominal infections caused by Gram-negative pathogens [14,15]. In both studies, CAZ-AVI was effective and well tolerated, with a safety profile similar to that of ceftazidime alone. The most frequently observed side effects were diarrhea, nausea and vomiting. 

There are no safety and efficacy data regarding the administration of CAZ-AVI in infants younger than 3 months of age, except for a few case reports [6,16,17,18,19,20,21,22]. 

Herein, we present the case of a 24-day-old neonate with pan-drug-resistant (PDR) *Klebsiella pneumoniae* successfully treated with the off-label use of CAZ-AVI, as a salvage therapy, born to a mother with an unknown maternal/gestational history and no routine prenatal care.

## 2. Case Presentation

A male infant, born at a gestational age of 36 weeks (3220 g b.w.), was admitted to the academic NICU Level III in the Pediatric Hospital “Iv. Mitev”, Sofia, Bulgaria, having been transferred from a secondary maternity hospital when he was at 24 days of life (DOL 24). 

The baby was born via spontaneous vaginal delivery with signs of dyspnea and moderate respiratory distress. Apgar scores were 5, 5 and 5 at 1, 3 and 5 min, respectively. Initial resuscitation, with nasal intermittent positive pressure ventilation (IPPV) for 30 min, was performed and empirical intravenous therapy was started with cefazolin (25 mg/kg/12 h), amikacin (7.5 mg/kg/12 h), fluconazole (6 mg/kg load, then 3 mg/kg once daily), meropenem (50 mg/kg/day divided every 8 h) and vancomycin (40 mg/kg/day divided every 8 h). Enteral feeding was initiated, however, with poor weight gain. Laboratory results revealed elevated C-reactive protein (55 mg/L) and severe metabolic acidosis. In the following days, intravascular coagulation was present with pulmonary hemorrhage, thrombocytopenia and coagulopathy. The infant also received multiple blood products without improvement. Blood culture was negative. The screen for genetically inherited metabolic disorders revealed no abnormalities. Clinical and laboratory tests revealed significant inflammatory activity (WBC 29.59 × 10^9^/L, HGB 128 g/L, HCT 37.8%, GGT 728 U/L, CRP 3.5 mg/L). Urinalysis showed proteins 2+, bilirubin 1+, urobilinogen 3.2 µmol/L, erythrocytes 2+. In addition, on DOL 14, projectile vomiting and diarrhea had started. An umbilical vein catheterization (UVC) was placed and the patient was switched to parenteral nutrition. Despite all the efforts made following accepted protocol the patient’s condition deteriorated (CRP 37.6 mg/L on DOL 21) and the infant (DOL 24) was referred to a multidisciplinary academic NICU level III. 

On admission (7 April 2022; DOL 24; body weight 2480 g), the patient was mildly hypotonic, pale with decreased muscle and neurological tone/activity, acrocyanotic, normothermic and had cold extremities. Examination revealed: heart rate 140/min, blood pressure 86/49 mm Hg, respiratory rate 55/min. Breathing was bilateral with mild dyspnea. One apneic episode was registered, on admission, and the patient was put on nasal IPPV. CRP was 180.54 mg/L (normal value < 5 mg/L). He presented with mixed (metabolic and respiratory) acidosis (pH 7.1, pCO_2_ 80 mmHg, HCO_3_ 19 mmol/L, BE 5.7 mmol/L) that was corrected. Cardiovascular and per abdomen examinations produced normal results. 

Urine and blood cultures were collected before the start of an empiric antibiotic treatment with intravenous imipenem/cilastatin (25 mg/kg every 8 h) and fluconazole (3 mg/kg daily). On the day after admission, the child was in a severely impaired general condition, febrile (38.4 °C). After consultation with a gastroenterologist, vancomycin (15 mg/kg every 8 h) and metronidazole (7 mg/kg every 8 h) were added to the empiric antibiotic treatment. Thrombocyte concentrate was also infused, due to thrombocytopenia (28 × 10^9^/L). Chest X-ray, transfontanellar and abdominal ultrasound were normal. Vitals, electrolytes, hepatic enzymes and blood gases were monitored as per typical NICU practice. 

On the 4th day of admission (DOL 28), urine culture results turned out to be negative, but the hemoculture was positive for pan-drug-resistant (PDR) *K. pneumoniae* ss. pneumoniae, identified by the automated VITEK^®^ 2 system. The microorganism was susceptible only to CAZ-AVI and resistant to amikacin, amoxicillin-clavulanate, ampicillin, ampicillin-sulbactam, cefepime, ceftazidime, ceftriaxone, cefuroxime, cephalexin, ciprofloxacin, colistin, meropenem, piperacillin-tazobactam and trimethoprim-sulfamethoxazole (Table 1). 

Antibiotic susceptibility testing was carried out using the Kirby–Bauer disk diffusion method, using a commercially available disc (BD BBL™ Sensi-Disc™) according to EUCAST guidelines [23]. As a result, CAZ-AVI therapy commenced based on the sensitivity profile of the blood culture result, as the only therapeutic option for our patient. A dose of 40 mg/kg (based on the dose of ceftazidime) every 8 h intravenously was determined, according to dose recommendation in the SmPC of Zavicefta^®^ [13] for the lowest age group of 3 months of age.

On 12th April (2nd day of CAZ-AVI therapy), enteral nutrition was initiated with good tolerance. Hemotransfusion was performed due to HGB 78 g/L and HCT 36.8%. For the next 3–4 days, the child continued to be in an impaired general condition, with edema, grayish skin, subfebrile and greenish stools containing mucus and blood. Due to persistent thrombocytopenia, 50 mL platelet concentrate “A” Rh(+) was infused daily, starting from the 2nd day of admission, for 6 consecutive days, until 4th day of CAZ-AVI therapy.

On 15 April 2022 (5th day of CAZ-AVI therapy), due to persistent, green-stained diarrhea with mucus, imipenem/cilastatin and metronidazole doses were increased to 30 mg/kg every 8 h and 8 mg/kg every 8 h, respectively. Fecal culture results were negative. A second blood culture was taken and two days later reported as negative.

On the 8th day of CAZ-AVI therapy (DOL 35) the child showed clinical signs of improvement, with mild dyspnea, pale-pink skin, normothermic, yellowish stools without mucus. A second blood transfusion was performed due to HGB 85 g/L and HCT 28.7%.

The patient’s condition gradually improved in the following days and a normalization of the CRP value was noted. On the 10th day of CAZ-AVI therapy, imipenem/cilastatin and metronidazole were discontinued and on the 15th day, vancomycin was also discontinued. On the 14th day of CAZ-AVI therapy, oxygen support was suspended and for the first time, normal stool was noted. CRP was in the normal range (3.12 mg/L). On the 16th day of CAZ-AVI therapy, a stable general condition, good enteral feeding with 60–70 mL Aptamil ADC (hypoallergenic formula), normal bowel movements and stool, heart rate 130/min and SaO_2_ 97–100% were reported. The patient demonstrated steady weight gain.

On 28 April 2022, CAZ-AVI (after 17 days of treatment) and fluconazole infusion therapy were discontinued. In the following days, the child was eating well, condition was stable, the abdomen was soft and defecation was normal. There were no abnormalities in biochemistry. The patient was discharged home on 5 May 2022, after 52 days of hospital stay, with a body weight of 3020 g. A year later, the patient was healthy with normal cognitive development.

## 3. Discussion

Clinical isolates of *K. pneumoniae* are generally resistant to a wide range of antibiotics [24,25]. High percentages of resistance in *K. pneumoniae* are of particular concern especially in the southern and eastern parts of the EU [24]. The percentage of *K. pneumoniae* cases that were combined resistant to third generation cephalosporins, fluoroquinolones and aminoglycosides, in 2021, was 59.9% and 21.2% for Bulgaria and EU/EEA, respectively [26]. One of the main reasons for AMR is antibiotic consumption and Bulgaria is one of the European countries with a very high total consumption of antibiotics (24.4 DDD/1000 inhabitants/day for 2021) [27,28,29]. *K. pneumoniae* is also among the most common Gram-negative bacteria isolated to the NICU and one of the leading causes of morbidity and mortality [30,31]. According to the Surveillance Atlas of Infectious Diseases, Bulgaria is one of the leading countries which detect resistant *K. pneumoniae*, *Escherichia coli* and *Acinetobacter* spp. isolates both in total and among children aged 0–4 years old (Table 2) [32].

We present the case of a 24-day-old infant transferred to a NICU level III from a secondary maternity hospital due to the deterioration of his general condition. Blood culture revealed PDR *K. pneumoniae*, resistant to β-lactams, aminoglycosides, tetracyclines, carbapenems, TMP-SMZ and colistin. The isolate was sensitive only to CAZ-AVI and thus this represented the only treatment option for the patient. NICU patients are particularly susceptible to microbial infections and very often exposed to many drugs early in life [33]. Our patient received five antimicrobial agents (cefazolin, amikacin, fluconazole and meropenem) before admission and four others (imipenem/cilastatin, metronidazole, vancomycin and fluconazole) after the initiation of the CAZ-AVI therapeutic regimen. Microbiologic and clinical responses were observed 5 days and 8 days after the initiation of CAZ-AVI therapy, respectively.

A search in PubMed for the literature on “ceftazidime-avibactam” and “neonates” resulted in seven relevant publications [16,17,18,19,20,21,22] (Table 3).

Due to the extremely limited experience of CAZ-AVI use in our NICU setting, we referred to the SmPC to closely monitor possible adverse drug reactions (ADRs). Blood electrolytes and urine analysis were monitored daily. Generally, CAZ-AVI has a favorable risk–benefit profile [34]. In our case, the patient tolerated CAZ-AVI very well with no severe adverse events detected. The patient was subfebrile on the 5th day of CAZ-AVI therapy, which was successfully treated with paracetamol. The patient was found to have thrombocytopenia the day after admission to NICU when vancomycin and metronidazole were initiated. The thrombocytopenia responded poorly to thrombocyte concentrate infusions and several daily infusions were performed (from the 2nd day of admission until the 4th day of CAZ-AVI therapy). From the 9th day until two days after cessation of CAZ-AVI therapy, hepatosplenomegaly was detected. Coskun and Atici [19] reported glycosuria on the 3rd day of CAZ-AVI therapy; Iosifidis et al. [17] reported hypomagnesemia in two neonates and hyperbilirubinemia in one neonate; and Nascimento et al. [21] reported abdominal distension and hypokalemia. Esposito et al. [18] reported thrombocytopenia as we also observed in our patient. However, it should be noted that, in our case, the thrombocytopenia was observed before the start of CAZ-AVI therapy, with no cause found. Ji et al. [20] did not report any adverse effects. One of the reported patients of Asfour et al. [16] died, despite the effectiveness of CAZ-AVI in eradicating *K. pneumoniae* and the negative blood culture on day 4 of the CAZ-AVI therapy. The death was probably due to prematurity, sepsis and/or chronic lung disease.

What we can learn from the article of Asfour et al. [16] is that in the two presented cases of premature-born neonates, CAZ-AVI was administered in a dose of 62.5 (50/12.5) mg/kg/dose every 8 h), which is higher than recommended [13]. In the review of Simeoli et al. [33], a dosage of 10–40 mg/kg every 8 h for those ≥3 to 6 months old with a creatinine clearance >50 mL/min/1.73 m^2^ was suggested. An intriguing case has been reported by Nascimento et al. [21], where CAZ-AVI was initiated on day 46 and discontinued on day 60 after a negative blood culture. However, on day 75 urine culture again revealed ESBL- and carbapenemase-producing *K. pneumoniae*, while blood culture remained negative. At that time, the patient had already been treated with ciprofloxacin, which continued for 10 days. The urine culture results were negative on day 83. It is interesting to note that an antibiotic sensitivity test, performed on day 46, detected *K. pneumoniae* resistant to ciprofloxacin.

## 4. Conclusions

### 4.1. Strengths

The treatment of PDR infections in NICUs is a great matter of concern. Intensive care procedures and multidrug therapies are crucial for case management. Off-label CAZ-AVI was initiated as a salvage therapy in a 28-day-old male with bacteremia, caused by proven PDR *K. pneumoniae* ss. pneumoniae. The CAZ-AVI therapy continued for 17 days with a dose of 40 mg/kg every 8 h (based on the dose of ceftazidime) and was very well tolerated with an uneventful recovery. A year later, the patient was healthy with normal cognitive development.

### 4.2. Lessons Learned

We strictly followed the recommended SmPC dose of CAZ-AVI, however miscalculation can occur, especially with products representing fixed-dose combinations, leading to treatment failure or the occurrence of ADRs. It is at the assessment of the medical staff, based on the specificity of the case, whether to follow the manufacturer’s instructions exactly. Available data (7 publications, n = 22 neonatal patients) suggest that CAZ-AVI is well tolerated. However, controlled, randomized clinical trials are needed to highlight the risks and benefits of CAZ-AVI in this unique population. Fortunately, no serious adverse drug reactions were observed in the reported cases. Since data on the use of newly licensed antibiotics in this age group are extremely limited, it is clinical expertise that can be relied on when determining the best treatment approach for neonates. This is particularly important as therapeutic options in NICUs are limited and clinical presentations are very diverse, while the patients are extremely immature and fragile.

### 4.3. Limitations

The clinical characteristics, laboratory examination, treatment and follow-up of the case were retrospectively analyzed. Since our patient received five antibiotics before and four others after admission, this limits our ability to evaluate CAZ-AVI treatment as if it had been applied as a stand-alone therapy. This may have led to a potentially biased interpretation of the data and limits our ability to draw definite conclusions.

## Figures and Tables

**Table 1:** Antibiotic susceptibility profile of *K. pneumoniae* ss. pneumoniae, identified by automated VITEK^®^ 2 system, according to EUCAST breakpoints [23].

Antibiotics	MIC (mg/L)/Zone Diameter (mm)	Result
Amikacin	>8/<18	Resistant
Amoxicillin-clavulanate	>8/<19	Resistant
Ampicillin	>8/<14	Resistant
Ampicillin-sulbactam	>8/<14	Resistant
Cefepime	>4/<24	Resistant
Ceftazidime	>4/<19	Resistant
**Ceftazidime/avibactam**	**≤8/>13**	**Susceptible**
Ceftriaxone	>2/<22	Resistant
Cefuroxime	>8/<19	Resistant
Cephalexin	>16/<14	Resistant
Ciprofloxacin	>0.5/<22	Resistant
Colistin	>2	Resistant
Meropenem	>8/<16	Resistant
Piperacillin-tazobactam	>8/<20	Resistant
Trimethoprim-sulfamethoxazole	>4/<11	Resistant

**Table 2:** Resistant *K. pneumoniae*, *E. coli* and *Acinetobacter* spp. isolates in total and among age 0–4.

Microorganism	Combined Resistance to	Total	Age 0–4
*Klebsiella pneumoniae*	3rd generation cephalosporins, fluoroquinolones and aminoglycosides	59.9%	50%
*Acinetobacter* spp.	Fluoroquinolones, carbapenems and aminoglycosides	71.9%	14.3%
*Esherichia coli*	3rd generation cephalosporins, fluoroquinolones and aminoglycosides	14.8%	11.1%

**Table 3:** Analysis of published cases using CAZ-AVI in neonates.

Author	Patient Age and Weight at Birth	Diagnosis	Culture	Notes	CAZ-AVI Dose and Duration of Therapy	Outcome
Asfour et al., 2022 [16]	Preterm female (27 gestational age) 920 g	Bacteraemia and meningitis	CSF (*K. pneumoniae*, sensitive to colistin, CAZ-AVI and fosfomycin)	Initial colistin therapy was ineffective	62.5 mg/kg/dose every 8 h for 21 days	cured
	Preterm female (28 gestational age) 925 g	Bacteraemia	Blood (*K. pneumoniae*, sensitive to meropenem and amikacin)	Initial meropenem therapy was ineffective	62.5 mg/kg/dose every 8 h for 4 days and 62.5 mg/kg/dose every 24 h (for 1 day)	died
Iosifidis et al., 2019 [17]	8 patients (median age 53 days, range from 13 days to 4.5 years; gestational age, 25–37 weeks)	Bloodstream infections (8 courses), central nervous system infections (2 courses) and urinary tract infection (1 course)	Microbiologically proven XDR or PDR *K. pneumoniae* in blood cultures, CSF, urine and rectal swab	All patients were critically ill and received other antibiotics prior to and concomitantly with CAZ-AVI	62.5 mg/kg/dose every 8 h for 4 to 38 days (median 14 days) In one case of febrile UTI due to renal insufficiency and CVVH, the dose was given at 50% of the recommended dose every 8 h.	cured
Esposito et al., 2019 [18]	Preterm male, 20-day old 860 g	Bacteremia and meningitis	Sensitive to CAZ-AVI, colistin, phosphomycin and tigecycline	Initial therapy with colistin, rifampicin, gentamicin, phosphomycin and meropenem was ineffective	75/20 mg/kg t.i.d. IV, but after 8 days decreased to 25/6.5 mg t.i.d. IV for 25 days. Due to recurrent infection, CAZ-AVI (25/6.5 mg t.i.d. IV) was administered for another 22 days, plus phosphomycin (45 mg/kg t.i.d. IV) and meropenem (40 mg/kg t.i.d. IV)	cured
Coskun & Atici., 2020 [19]	Preterm male (27 gestational weeks) 1000 g	UTI	On day 6, blood and urine culture—carbapenem-resistant *K. pneumoniae,* sensitive to colistin; second blood culture on day 25—resistant to colistin, but sensitive to CAZ-AVI and tigecycline	Initial meropenem, colistin and vancomycin therapy was ineffective	50 (40/10) mg/kg/dose every 8 h for 10 days	cured
Ji et al., 2021 [20]	81-day male	Osteomyelitis	Blood and bone marrow fluid culture (*K. pneumoniae*, sensitive to colistin, CAZ-AVI, tigecycline and TMP-SMZ)	Initial imipenem/cilastatin and fosfomycin therapy was ineffective	CAZ-AVI 50 (40/10) mg/kg IV every 8 h for 14 days	cured
Nascimento et al., 2023 [21]	Preterm male (29 weeks gestational age) 830 g	Bloodstream infection	Sensitive to CAZ-AVI, amikacin and colistin	ampicillin, gentamicin, fluconazole, meropenem, amphotericin B deoxycholate, vancomycin, linezolid, polymyxin B, micafungin and phenobarbital	CAZ-AVI 50 (40/10) mg/kg IV q8h for 2 days; next two days adjusted to peritoneal dialysis to 23.75 mg/kg IV q48 h; followed by 50 mg/kg IV q8h	cured
Marino et al., 2023 [22]	Eight preterm patients (3 males, 5 females)	Blood cultures	Sensitivity to CAZ-AVI and several other antibiotics	Due to the worsening of neonates’ clinical status and despite empirical broad spectrum antibiotic therapies, CAZ-AVI was initiated	50 (40/10) mg/kg/dose every 8 h for medium 14 days	cured

Abbreviations: ETA (endotracheal aspirate), CSF (cerebrospinal fluid), CVVH (continuous venous-venous hemofiltration), UTI (urinary tract infection), XDR (extensive drug resistance), PDR (pan drug resistance), CAZ-AVI (ceftazidime/avibactam), TMP-SMZ (trimethoprim-sulfamethoxazole).

## Data Availability

Additional clinical and laboratory findings during CAZ-AVI therapy are available on request from the corresponding author.
